# Nuclear myosin/actin-motored contact between homologous chromosomes is initiated by ATM kinase and homology-directed repair proteins at double-strand DNA breaks to suppress chromosome rearrangements

**DOI:** 10.18632/oncotarget.24434

**Published:** 2018-02-07

**Authors:** Viktoria N. Evdokimova, Manoj Gandhi, Alyaksandr V. Nikitski, Christopher J. Bakkenist, Yuri E. Nikiforov

**Affiliations:** ^1^ Department of Pathology, University of Pittsburgh, Pittsburgh, PA, 15213, USA; ^2^ Department of Radiation Oncology and Pharmacology and Chemical Biology, University of Pittsburgh, Pittsburgh, PA, 15232, USA

**Keywords:** chromosome, double-strand break, ATM, nuclear motors, DNA repair

## Abstract

We provide evidence for a mechanism of DNA repair that requires nuclear myosin/actin-dependent contact between homologous chromosomes to prevent formation of chromosomal rearrangement in human cells. We recently showed that DNA double strand breaks (DSBs) induced by γ-rays or endonucleases cause ATM-dependent contact formation between homologous chromosomes at damaged sites of transcriptionally active chromatin in G_0_/G_1_-phase cells. Here, we report that the mechanism of contact generation between homologous chromosomes also requires homology-directed repair proteins, including BRCA1, RAD51 and RAD52, and nuclear myosin/actin-motors. Moreover, inhibition of ATM kinase or deficiency in nuclear actin polymerization causes carcinogenic *RET/PTC* chromosome rearrangements after DSBs induction in human cells. These data suggest that DSBs in transcriptionally active euchromatin in G_0_/G_1_-phase cells are repaired through a mechanism that requires contact formation between homologous chromosomes and that this mechanism is mediated by HDR proteins and nuclear myosin/actin motors.

## INTRODUCTION

Mechanisms that ensure genome stability were essential for the origin of species and homeostasis in metazoans. DNA double-strand breaks (DSBs) which are induced by reactive oxygen radicals generated by oxidative phosphorylation and ionizing radiation (IR) are a particular hazard in mammalian cells as they are substrates for recombination events that can activate oncogenes leading to cancer. Two canonical DSB repair pathways exist in eukaryotic organisms. Non homologous end-joining (NHEJ) is an imperfect mechanism of repair wherein DSB ends are trimmed and then the ends are ligated together leading to a loss of genetic material at the site of the break. Homologous recombination is a mechanism of repair that uses a sister chromatid as a template to correct a DSB without loss of genetic material. Since this canonical mechanism of homologous recombination requires the co-localization of sister chromatids, it is generally associated with DNA replication forks in S phase or with G2 phase cells. However, observations that homologous chromosomes make contact after the induction of DSBs in G_0_/G_1_ cells, including mature thyroid cells [1, 2], led us hypothesize that a mechanism of homologous recombination that uses a homologous chromosome as a template may exist in cells when there is no sister chromatid template.

Several additional lines of evidence point to a mechanism that generates contact between homologous chromosomes after the induction of a DSB. Live cell imaging of diploid budding yeast revealed that endonuclease-induced DSB end in a red fluorescent protein-marked chromosome is rapidly repaired using the yellow fluorescent protein-marked homologous chromosome during S-phase [3]. Following DSB induction, the cleaved chromosome was observed moving through ∼30% of the nuclear volume, rather than 3% prior to DSB induction. Contact between homologous chromosomes was coincident with Rad52 foci that identify sites of strand exchange between chromatids or chromosomes. Homology-directed repair (HDR) was inferred from the observation that Rad52 foci disassembled before the homologous chromosomes separation. Increased mobility of an endonuclease-induced DSB end was also observed in haploid yeast [4]. The increased mobility of the DSB end required Rad51, a key recombinase protein that identifies sequence homology and pairs sister chromatids, Mec1, an apical DNA damage signaling kinase, and Rad9, a checkpoint protein. The increased mobility of the DSB end did not require the downstream kinase Rad53, suggesting that in yeast an apical DNA damage signaling kinase, but not checkpoint activation *per se* was required for the increased mobility.

In mammalian cells, increased mobility of γ-particle-induced DSB ends and uncapped telomeres were observed and this was dependent on 53BP1, the homologue of yeast Rad9 [5–7]. In contrast, limited mobility of DSB ends was observed after application of ultrasoft-X-ray or single endonuclease [8–10]. Allowing one explanation for this apparent discrepancy, we showed that contact between homologous chromosomes induced by either γ-rays or an endonuclease was limited to sites of DSBs that arose in transcriptionally active chromatin in human cells [1, 2]. These data are generally supported by the finding that DSBs in transcriptionally active chromatin are preferentially repaired by homologous recombination in human cells [11]. However, factors required for homologous chromosomes mobility, their contact initiation or consequences of this failure remain largely unknown.

Increased mobility of endonuclease-induced DSB ends in telomeres in ALT (alternative lengthening of telomeres) positive G_1_-phase cancer cells has been reported [12]. The introduction of a DSB in an ALT-telomere induced RAD51-mediated telomere-telomere contact, which was associated with homology-directed telomere synthesis. DSBs induced in transcriptionally active rDNA repeats in nucleoli using either an endonuclease or CRISP/Cas9 targeting increased the mobility of DSB ends of rDNA [13]. The introduction of a DSB in transcriptionally active rDNA repeats induced the migration of the site of the DSB from the nucleolar interior to anchoring points at the nucleolar periphery in G_1_-, S- and G_2_-phase cells. The migration was ATM kinase-dependent and RAD51 foci co-localized with DSBs in rDNA at the nucleolar periphery. These data suggest that the mobility of ALT-telomere and nucleolar rDNA is increased after the induction of a DSB and that proteins required for homology-directed repair accumulate at these DSB ends in G_1_-phase cells. In addition to ATM and RAD51, the long-range directional movement of an interphase chromosome site may be dependent on nuclear myosin I (NMI) and actin nuclear motors [14].

Here, we show that contact between homologous chromosomes after induction of DSBs in transcriptionally active chromatin in G_0/_G_1_-phase human cells requires homology-directed repair (HDR) but not non homologous end join (NHEJ) proteins. Furthermore, we demonstrate that it requires nuclear myosin/actin-motors. Finally, we show that ATM kinase inhibition or genetic inactivation of nuclear actin induce carcinogenic *RET/PTC* chromosome rearrangements in human cells.

## RESULTS

### Contact between homologous chromosomes at sites of DSBs induced in genes requires HDR proteins

Previously, we showed contact formation between homologous chromosomes at sites of DSBs in quiescent cells in transcriptionally active chromatin [2]. This contact formation required ATM, an apical DNA damage signaling kinase that is activated at DSBs and necessary for homology-directed DNA repair (HDR) pathways [15, 16]. We hypothesized that contact formation between homologous chromosomes after induction of DSBs in transcriptionally active chromatin also required HDR proteins.

**Figure 1:**
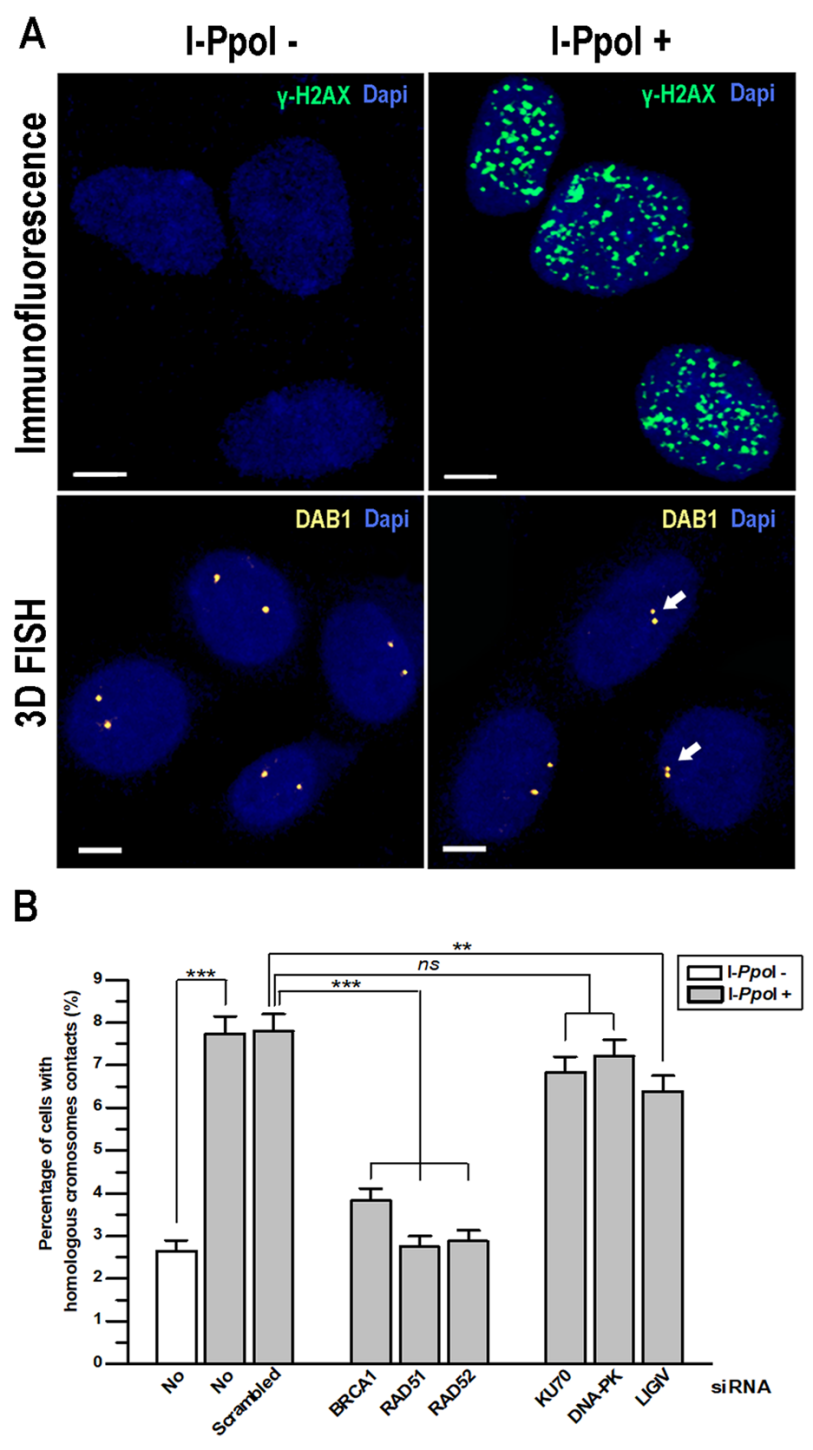
Contact formation between homologous chromosomes at DSBs in *DAB1* and *GRIP1* genes requires HDR proteins TPC1/I-PpoI cells were treated with 4-OHT for 6 h to induce translocation of the ER-I-PpoI fusion protein into the nucleus and generate DSBs at specific sites. **(A)** Efficiency of DSB induction by I-PpoI in TPC1/I-PpoI cells was confirmed by immunofluorescence with anti-γH2AX antibodies. The intranuclear localization of I-PpoI-sensitive *DAB1* gene regions of homologous chromosomes without and after induction of DSBs by I-PpoI was determined with 3D FISH. Arrows identify contact formation between homologous chromosomes. Scale bar - 5 μm. **(B)** Graph showing the increase in contact formation between homologous chromosomes at *DAB1* and *GRIP1* genes after induction of DSBs by I-PpoI. Knockdown of HDR proteins (BRCA1, RAD51 and RAD52) by siRNA abolished contact formation between homologous chromosomes. Data for *DAB1* and *GRIP1* regions are combined and presented as mean ± SEM; **, *P* < 0.001; ***, *P* < 0.0001.

To test this hypothesis, we generated human thyroid cancer TPC1/I-PpoI cells stably expressing HA-ER-I-PpoI fusion protein which translocates into nucleus and induces DNA DSBs at specific restriction sites upon induction with 4-hydroxytamoxifen (4-OHT) [17, 18]. Expression of genes required for HDR (*BRCA1*, *RAD51* and *RAD52*) and for NHEJ (*KU70*, *DNA-PK* and *LIGIV*) was knocked down in TPC1/I-PpoI cells using siRNA (Supplementary Figure 1). I-PpoI-specific DSBs were then induced by treatment with 4-OHT, that was confirmed by γH2AX immunofluorescence. Frequency of contact formation between homologous chromosomes was analyzed at two I-PpoI-sensitive sites in the transcriptionally active *DAB1* and *GRIP1* genes in G_0_/G_1_-phase cells using 3D FISH (Figure 1A).

Our analysis revealed that frequency of contact between homologous chromosomes at *DAB1* and *GRIP1* genes upon induction of DSBs increased from baseline level of 2.6% to 7.7% and 7.8% in intact and scramble siRNA treated cells, respectively. This finding is concordant with previous data demonstrated that the I-PpoI restriction endonuclease cuts approximately 10% of sequence specific sites in a single cell [2, 19]. When any of HDR genes (*BRCA1*, *RAD51* and *RAD52*) expression was knocked down, the contact between homologous chromosomes was reduced to the baseline level. However, when the NHEJ genes were silenced, the frequency of I-PpoI-induced homologous chromosome contact was either unaffected after knocking down *DNA-PK* and *KU70* or slightly reduced after knocking down *LIGIV* (Figure 1B).

Thus, the HDR factors BRCA1, RAD51 and RAD52, but not NHEJ factors DNA-PK and KU70 are required for DSBs-induced contacts formation between homologous chromosomes at the transcribed *DAB1* and *GRIP1* genes in G_0_/G_1_-phase cells.

### Rad51 co-localizes with sites of contact between homologous chromosomes at DSBs induced in genes in G_0_/G_1_-phase cells

**Figure 2:**
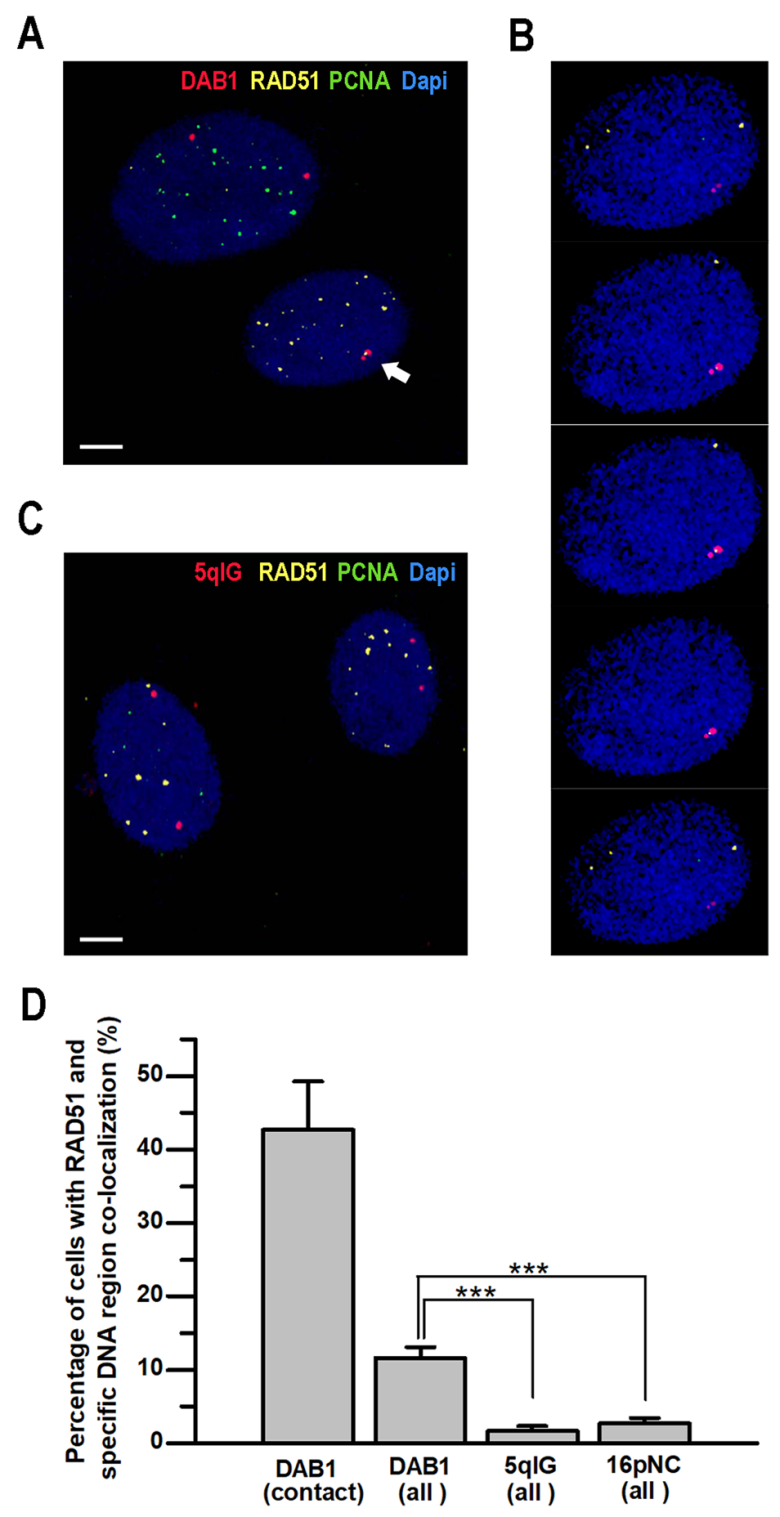
Co-localization of RAD51 with sites of contact between homologous chromosomes after induction of DSBs in G_0_/G_1_-phase cells TPC1/ I-PpoI cells were treated with 4-OHT for 6 hours prior to 3D immuno-FISH analysis. **(A)** Representative 3D immuno-FISH confocal image showing co-localization of RAD51 with homologous chromosomes contact (arrow) at transcriptionally active *DAB1* gene in G_0_/G_1_-phase cells (lower nucleus). S-phase cells with characteristic focal pattern of PCNA staining (upper nucleus) were excluded from analysis. **(B)** 3-D co-localization of the RAD51 with *DAB1* signals was confirmed by sequential analysis of the Z-stacked images. **(C)** 3D immuno-FISH with probe for the I-PpoI-sensitive 5qIG intergenic region did not reveal notable co-localization of 5qIG and RAD51 signals. Scale bar - 5 µm. **(D)** Graph showing higher frequencies of RAD51 co-localization with transcriptionally active *DAB1* gene in G_0_/G_1_-phase cells, especially at sites of inter-chromosomal contacts, compared to the silent intergenic 5qIG or I-PpoI –insensitive 16pNC chromosomal regions. Data are presented as mean ± SEM;***, *P* < 0.0001.

To further confirm the role of HDR factors in contact formation between homologous chromosomes after induction of DSBs, we performed 3D immuno-FISH with DNA probes for the I-PpoI-sensitive regions of *DAB1* gene and intergenic 5qIG locus or for the I-PpoI-insensitive 16pNC non-coding region. Additionally, antibodies against RAD51 and PCNA were used. Only G_0_/G_1_-phase cells showing diffuse/homogenous PCNA staining were analyzed. 3D immuno-FISH showed that after induction of DSBs, RAD51 was co-localized with 43% of contacts between homologous chromosomes at *DAB1* gene (Figure 2). RAD51 foci were not expected in 100% of contacts since part of homologous chromosomes might be in contact after the dissociation of the RAD51 filaments. Regardless of the chromosome contact, RAD51 foci were co-localized with the transcriptionally active *DAB1* gene in 11.7% of G_0_/G_1_-phase cells. In contrast, RAD51 was co-localized with I-PpoI-sensitive intergenic 5qIG locus only in 1.7% of cells (Figure 2C, 2D). This was similar to the baseline level of spontaneous RAD51 foci co-localization with anti-16pNC FISH probes (Figure 2D). These data reveal that RAD51 co-localizes with contacts between homologous chromosomes at sites of DSBs induced in transcriptionally active chromatin in G_0_/G_1_-phase cells.

### Contact between homologous chromosomes at sites of DSBs induced in genes in G_0_/G_1_-phase cells requires nuclear myosin I and actin nuclear motors

Based on the previous findings implicating NMI and actin in the directional movements of interphase chromosomes, we investigated the role of these proteins in contact formation between homologous chromosomes at sites of DSBs induced in genes. For this, NMI was knocked down in TPC-1/I-PpoI cells by treatment with specific siRNA (Supplementary Figure 1). Alternatively, the function of actin was blocked using two drugs that affect F-actin polymerization, jasplakinolide [20] and latrunculin B [21].

**Figure 3:**
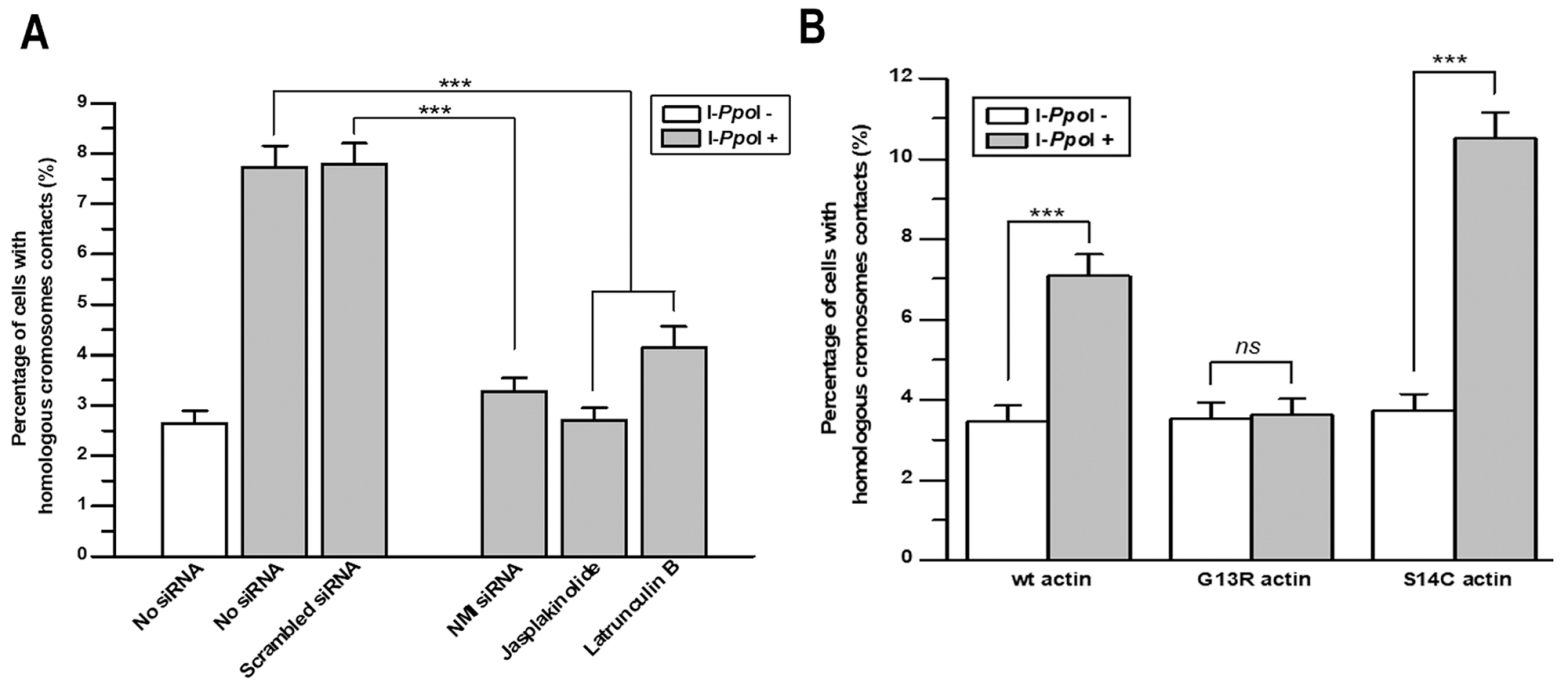
Nuclear myosin I and actin are required for contact formation between homologous chromosomes at sites of DSBs in *DAB1* and *GRIP1* genes **(A)** Knockdown of NMI using siRNA for 72 h or disruption of F-actin polymerization with jasplakinolide or latrunculin B for 2 h decreased the frequency of contact formation between homologous chromosomes at DSBs in *DAB1* and *GRIP1* genes. **(B)** Graph showing that polymerization deficiency of G13R-mutant actin reduced the frequency of contact formation between homologous chromosomes. In contrast, S14C-actin, which promotes filament polymerization, increased contact formation between DSBs sites of homologous chromosomes. Data are presented as mean ± SEM; ***, *P* < 0.0001.

As shown before, contacts between homologous chromosomes at sites of DSBs induced in the *DAB1* and *GRIP1* genes were identified in 7.4% and 7.8% of intact and scramble siRNA treated cells, respectively. Upon knock-down of NMI, the frequency of the contact between homologous chromosomes decreased to 3.2%, close to the baseline observed without DSBs induction. Similar decrease was revealed after treatment with jasplakinolide and latrunculin B (Figure 3A).

To further explore the role of nuclear motors in homologous chromosomes contact formation, we transfected TPC-1/I-PpoI cells with expression plasmids for wild-type actin, G13R-mutant actin that is deficient in F-actin polymerization, and S14C-mutant actin that in opposite increases F-actin polymerization [14, 22]. In the absence of DSBs-induction, contacts between homologous chromosomes at the *DAB1* and *GRIP1* genes were identified in 3.5-3.7 % of cells expressing different types of actin. However, I-PpoI-induced homologous chromosome contact at *DAB1* and *GRIP1* was identified in 7.0% of cells expressing wild-type actin; in 3.6% of cells expressing G13R-mutant actin that is deficient in actin polymerization; and in 11.0% of cells expressing S14C-mutant actin that increases F-actin polymerization (Figure 3B). Thus, three different experimental approaches demonstrate that NMI and proper polymerization of F-actin are required for contact formation between homologous chromosomes at the sites of DSBs in active genes in G_0_/G_1_-phase cells.

### ATM kinase activity and F-actin polymerization are required for chromosome stability after the induction of DSBs

Since ATM kinase activity and myosin/actin nuclear motors are required for contact between homologous chromosomes at DSBs in transcriptionally active chromatin, we hypothesized that inhibition of ATM kinase or F-actin polymerization would prevent the formation of homologous chromosomes contact at sites of DSBs, thereby promoting breaks misrepair and formation of chromosome rearrangements. To test this hypothesis, we used an assay that detects the formation of endonuclease-induced *RET/PTC* rearrangements [23, 24]. Analyzed *RET/PTC1* and *RET/PTC3* are intrachromosomal inversions involving the *RET* and *CCDC6* or *NCOA4* genes, respectively, which are prevalent in thyroid cancers in individuals exposed to radiation after the Chernobyl nuclear accident [25]. Importantly, *RET/PTC* can be induced in G_0_/G_1_-phase thyroid cells *in vivo* by exposure to ionizing radiation or restriction endonucleases [23, 24]. The endonuclease PvuII induces DSBs in intron 11 of the *RET* gene, which lead to the formation of *RET/PTC* in HTori-3 human thyroid cell line [24].

**Figure 4:**
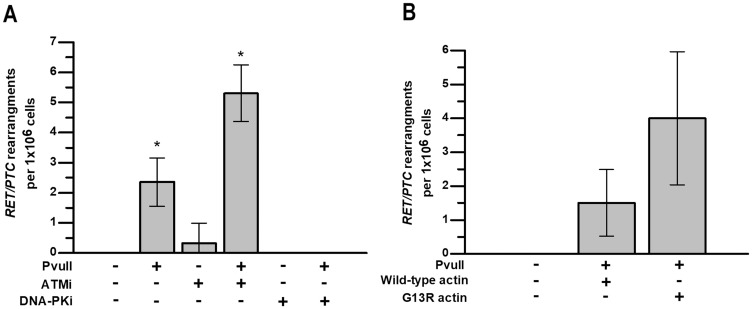
ATM activity and F-actin polymerization are required for chromosomal stability after induction of DSBs HTori-3 cells were treated for 4 h with ATM kinase inhibitor KU55933 or DNA-PK inhibitor NU7441 immediately after electroporation with PvuII restriction endonuclease. **(A)** Graph showing the effect of ATM and DNA-PK inhibition on the formation of *RET/PTC* rearrangements in HTori-3 cells with and without PvuII-induced DNA DSBs. **(B)** Graph showing that F-actin polymerization deficiency after introduction of G13R-mutated actin increased the frequency of *RET/PTC* rearrangements in HTori-3 cells treated with PvuII endonuclease. Data are presented as means with 95%CI; ^*^, *P* < 0.05.

First, we investigated how ATM kinase inhibition impacts the endonuclease-induced *RET/PTC* rearrangements. HTori-3 cells were electroporated with PvuII restriction enzyme and treated with the ATM inhibitor KU55933. DSB induction by PvuII was assessed by γH2AX immunofluorescence and 75-82% of cells electroporated with PvuII were positive for DNA damage, as compared to 5-6% of control cells (data not shown). PvuII-induced *RET/PTC* were observed in 2.4±0.4 cells per million in vehicle control, and this increased to 5.3±0.5 cells per million with ATM kinase inhibitor. Surprisingly, in the absence of PvuII, *RET/PTC* rearrangements were observed in 0.3±0.3 cells per million with ATM kinase inhibitor (Figure 4A). Thus, ATM kinase inhibition promotes *RET/PTC* rearrangements in human cells, even in the absence of an exogenous source of DSBs.

Next, we investigated how DNA-PK inhibition impacts endonuclease-induced DSB end-initiated *RET/PTC* rearrangements. It has previously been shown that *RET/PTC* fusions occur at sites where there are 3-4 bp of sequence homology between the genes [23], which is characteristic of NHEJ. To test whether DNA-PK activity is required for the rearrangement to occur, HTori-3 cells were electroporated with PvuII restriction enzyme and treated with the pharmacologic DNA-PK inhibitor NU7441. No rearrangements was detected in cells treated with combination of PvuII and DNA-PK inhibitor or with DNA-PK inhibitor alone (Figure 4A). This data, which is strikingly different to those obtained with the ATM kinase inhibitor, suggests that inhibition of DNA-PK, and therefore classical NHEJ, prevents the formation of *RET/PTC* rearrangements.

Finally, we investigated how inhibition of F-actin polymerization impacts endonuclease-induced DSB end-initiated *RET/PTC* rearrangements. For this, HTori-3 cells were transfected with vectors expressing either wild-type actin or G13R-mutated actin that is deficient in actin polymerization. PvuII-induced *RET/PTC* rearrangements were observed in 1.5±0.5 cells per million in cells expressing wild-type actin, and in 4±1 cells per million in cells expressing G13R-mutated actin (Figure 4B). The inhibition of F-actin polymerization in PvuII-electroporated HTori-3 human cells showed the tendency for the increase in frequency of *RET/PTC* rearrangements, however it did not reach the level of statistical significance.

## DISCUSSION

In this study, we show that contact between homologous chromosomes after induction of DSBs in transcriptionally active chromatin in G_0_/G_1_-phase cells requires HDR proteins as well as nuclear myosin/actin motors. Furthermore, we demonstrate that ATM kinase inhibition or F-actin polymerization deficiency induce carcinogenic *RET/PTC* chromosome rearrangements in human cells.

In addition to our previous study where we reported the role of ATM kinase activity in homologous chromosomes contact [2], using 3D FISH and immuno-FISH we demonstrate that BRCA1, RAD51 and RAD52 are also required to generate contact between homologous chromosomes after induction of DSBs in G_0_/G_1_-phase human cells. 3D FISH allows the direct enumeration of chromosomes and to exclude cells containing sister chromatids. In addition, 3D immuno-FISH with anti-PCNA antibody identifies G_0_/G_1_-phase cells on the basis of pan-nuclear PCNA staining. Work in yeast predicts that HDR proteins will be recruited to a DSB end in G_0_/G_1_-phase cells before its mobility is increased and contact between homologous chromosomes is generated [3, 4]. We found that RAD51 co-localizes with almost half of endonuclease-induced sites of contact between homologous chromosomes in G_0_/G_1_-phase cells. This suggest that the apical DNA damage signaling kinase- and HDR protein-dependent mechanism, that generates contact between homologous chromosomes after the induction of DSBs in G_0_/G_1_-phase cells, is conserved from yeast to human cells, although in yeast it is Mec1-dependent, and in humans it is ATM-dependent rather than ATR (Mec1)-dependent [3, 4]. We have previously shown that ATM kinase activity and active transcription are required to generate contact between homologous chromosomes induced by DSBs in transcriptionally active chromatin outside of the nucleolus [1, 2]. And it is consistent with the recent finding that ATM kinase activity is strictly required for the movement of DSBs in actively transcribed rDNA to the nucleolar periphery in G_0_/G_1_-phase human cells [13]. Thus, it appears that the mechanisms of DSB-induced mobility in transcribed rDNA and transcribed genes outside of the nucleolus are both dependent on ATM kinase and HDR proteins including RAD51.

A major mechanistic finding in this report, not been described in any other system to date, is that nuclear motors are required for contact between homologous chromosomes after the induction of DSBs in transcriptionally active regions, and these may play significant role in preserving genome integrity after DNA damage. Previous studies have shown that long-range directional movement of an interphase chromosome site is dependent on specific actin or nuclear myosin I (NMI) nuclear motors [14]. Our findings demonstrate that expression of mutant actin defective in polymerization as well as exposure to chemical inhibitors of nuclear motors, inhibits contact between homologous chromosomes after induction of DSBs in transcriptionally active chromatin.

To determine the functional significance of the ATM kinase- and nuclear motor-dependent mechanism that generates contact between homologous chromosomes after induction of DSBs in transcriptionally active chromatin, we enumerated carcinogenic chromosomal rearrangements using recently established assay that utilizes restriction enzymes to induce targeted DSBs in human thyroid cells HTori-3 [24]. Using this assay, we showed that ATM kinase activity is required for the repair of actively transcribed DNA, and ATM kinase inhibition can induce the *RET/PTC* carcinogenic rearrangements. Similarly, we observed that expression of mutant non-polymerizing actin, which also prevents homologous chromosomes contact, shows tendency to induce rearrangement in human cells. In summary, the results of this study reveal that contact between homologous chromosomes at the sites of DSBs in actively transcribed genes is dependent on HDR proteins and nuclear myosin/actin motors; functional ATM kinase and proper F-actin polymerization are required for prevention of carcinogenic chromosomal rearrangement generation upon induction of DNA DSBs in human cells.

## MATERIALS AND METHODS

### Cell lines

TPC-1 is a human cell line derived from a papillary thyroid carcinoma that expresses the *RET/PTC1* oncogene. We have previously confirmed that *DAB1* and *GRIP1* chromosome loci studied here are diploid in interphase TPC-1 cells [1, 2]. TPC1 cell line was propagated in DMEM media supplemented with 10% FBS. HTori-3 is a human normal thyroid cell line originally immortalized by SV40 transfection [26]. These cells are partially transformed but retain thyroid differentiation characteristics expressing sodium/iodide symporter and thyroglobulin [23, 24]. HTori-3 cell line was propagated in RPMI 1640 media supplemented with 10% FBS.

### Induction of DNA DSBs by I-PpoI in TPC-1 cells

To establish a cell line stably expressing I-PpoI, HA-ER-I-PpoI, retrovirus (gift of M. Kastan, Duke Cancer Institute, Durham, NC) was generated as described previously [27] and used to infect TPC-1 cells. Selection of TPC-1 cells expressing HA-ER-I-PpoI (TPC-1/I-PpoI) was done using supplementation of culture medium with 1 μg/mL puromycin for 2 weeks. To induce DNA DSBs, cells were treated with 1μM 4-hydroxytamoxifen (4-OHT) for 6 h [17, 28]. DSB induction was monitored by γH2AX immunofluorescence.

### γH2AX immunofluorescence

Immunofluorescence staining for γH2AX was performed to confirm formation of I-PpoI- and PvuII-induced DSBs in TPC-1/I-PpoI and Htori-3 cells, respectively. Cells cultured on coverslips were fixed with ice-cold 4% formaldehyde for 10 min followed by the permeabilization with 0.5%Triton X-100/PBS on ice for 5 min. After washing with PBS, cells were stained with staining solution (20 mM Tris, pH7.6, 137 mMNaCl,10% skimmed milk, and 0.5% Tween-20) containing anti-phosphorylated histone H2AX primary antibody (Upstate Biotechnology, Buffalo, NY, USA) at 1:1000 dilution for 2 h at 37°C. Coverslips were washed with PBS twice and incubated with Alexa488-labeled anti-mouse IgG (Molecular Probes, Eugene, OR, USA) in staining solution (1:200) for 1 h at 37 8C. After washing with PBS, samples were counterstained with DAPI. Cells with more than five γH2AX foci per nucleus were considered positive for I-PpoI- or PvuII-induced cleavage.

### Analysis of contact between specific regions of homologous chromosomes using three-dimensional FISH and immuno-FISH

To analyze homologous chromosomes contact, TPC/I-PpoI cells from each experiment were 3D fixed and processed to FISH with region-specific probes. Cells cultured in chamber slides from each experiment were subjected to 3D fixation with 4% paraformaldehyde for 10 minutes at 4°C, permeabilization on ice with 0.5% Triton X-100/PBS for 15 minutes followed by immersion into ice-cold 20% Glycerol/PBS for 30 min and 4-6 times freeze-thaw treatment in liquid nitrogen [29]. For FISH, 3D fixed slides were washed in 0,05% Triton X-100/PBS for 15 min at room temperature (RT) and pretreated with 100ug/ul RNase A in 1mM EDTA/PBS for 1 hour at 37°C, followed by post-fixation with 2% paraformaldehyde for 5 minutes at RT. After washing in 2XSCC slides were subjected to hybridization (73°C - 5minutes/37°C - 14-18 hours) with labeled probe in hybridization buffer (55%formamide, 10% dextran sulfate in 2xSCC, pH7.0). For FISH probes generation, RP11-328F17 + RP11-393123 (*DAB1* gene region), RP11-754J4 (*GRIP1* gene region), CH17-12M21 (5q15-q21 intergenic region) and RP11-513N24 (16q22 intergenic region) BAC clones (BAC/PAC Resources, Children’s Hospital, Oakland) were labeled by nick translation using d-UTP tagged Spectrum dyes (Abbott Laboratories) according to manufacture instructions. Post hybridization washes were performed with 50% formamide/2xSSC at 42°C and 2XSCC at 37°C followed by embedding with VECTASHIELD mounting medium with DAPI (Vector Laboratories).

Immuno-FISH, i.e. immunostaining followed by 3D FISH, was performed for the simultaneous detection of specific DNA targets and nuclear proteins [30, 31]. Mouse monoclonal antibodies for PCNA (Sigma-Aldrich, P-8825, 1:1500) and rabbit polyclonal antibody for RAD51 (Abcam, ab 63801, 1:100) were used. Briefly, cultured cells were fixed with ice-cold 4% PFA for 10 minutes and permeabilized on ice with 0.5% Triton X-100/PBS for 15 minutes followed by standard immunostaining with sequential incubations with primary and secondary antibodies. Immunostained slides were subjected to post-fixation with 2% PFA for 10 minutes at RT, equilibration with 20% Glycerol/PBS followed by 4-6 freeze-thaw cycles and FISH as described above.

Confocal microscopy was performed using a Leica SP5 TCS 4D confocal laser scanning fluorescence microscope using a 63×, 1.4 N.A. oil PlanApo objective. For each experimental condition, at least 1,000 nuclei of TPC/I*-*PpoI G_0_/G_1_-phase cells were analyzed. We identified and evaluated G_0_/G_1_-phase cells on the basis of their morphology and the unambiguous identification of 2 copies of each gene or diffuse PCNA staining. Nuclear boundaries were identified by DAPI staining, and image stacks were acquired with z steps of 0.13 μm. The digital image stacks were reconstructed using Velocity software (PerkinElmer). Site-specific probes in each nucleus were identified by using the appropriate filter cube, and signals were scored as positive for contact when the space between them was smaller than size of one signal.

### siRNA gene silencing and Quantitative qRT-PCR

Down-regulation of HDR pathway (*BRCA1*, *RAD51*, *RAD52*), NHEJ pathway (*KU70*, *DNA-PK*, *LIGIV)* and *NMI* genes expression in TPC1/I-PpoI cells was performed using siRNA pools (Dharmacon) according to manufacturer instructions. Change in expression levels of studied genes were analyzed using qRT-PCR with CyberGreen (ABI Biosystems) 72 hours after treatment with siRNAs. Data of *PGK* expression was used for normalization. Experiments were performed in triplicates.

### Chemical disruption of normal F-actin polymerization

To alter F-actin polymerization in TPC-1/I-PpoI cells they were treated with 200nM Latrunculin A or 100nM Jasplakinolide for 2 hours after DSBs induction with 4-OHT. Experiments were performed in triplicates.

### Chemical inhibition of ATM and DNA-PK

Htori-3 cells were treated with 10 μM ATM kinase inhibitor KU55933 or 5 μM DNA-PK inhibitor NU7441 for 4 hours immediately after electroporation with PvuII restriction enzyme. The efficacy of KU55933 and NU7441 in HTori-3 cells was determined by immunoblotting with anti-ATM (MAT3-4G10/8; Sigma-Aldrich), anti-phosphoserine-1981 ATM (EP1890Y; Epitomics), anti-DNA-PK (4062; Cell Signaling) and anti-phosphoserine-2056 (18192; Abcam) antibodies (data not shown). Experiments were performed in triplicates.

### Actin plasmids

Three expression plasmids (gift of Dr. A Belmont) coding different forms of actin were used: wild type actin, G13R actin that is deficient in F-actin polymerization and mutant S14C actin that increases F-actin polymerization [14, 22]. TPC1/I-PpoI cells were transfected with wild type, G13R or S14C actin coding plasmids; Htori-3 cells were transfected with wild type or G13R actin coding plasmids using Lipofectamine 2000 (Invitrogen) according to manufacturer protocol. After 2 weeks of selection with 500 μg/mL of G418, cells were subjected to the experiments with DSBs induction.

### PvuII-induced *RET/PTC* rearrangements in Htori-3 cells

Scheme of the detection of *RET/PTC* re-arrangements after introduction of DNA DSBs by PvuII is illustrated in Supplementary Figure 2. For each experiment 2×10^6^ of HTori-3 cells were electroporated with 25U of PvuII restriction enzyme (New England Bio Labs, Ipswick, MA, USA) at 50 mC charge (400 V and 125 mF) using Gene Pulser Xcell Electroporator (Bio-Rad) as previously described [32]. DSB induction was monitored by γH2AX immunofluorescence staining at 4 h post-electroporation. For the detection of PvuII-induced *RET/PTC* rearrangements the electroporated cells were seeded in 30 T25 flasks (6.6×10^4^ cells per flask). To sustain continuous growth, cells were transferred to T75 flasks and harvested when 90% confluence was reached. RNA was extracted from each flask using a Trizol reagent (Invitrogen) and mRNA was purified using Oligotex mRNA mini kit (Qiagen). Detection of *RET/PTC* was performed using RT followed by PCR with primers for *RET/PTC1* and *RET/PTC3;* and Southern blot hybridization of the PCR products with ^32^P-labeled oligonucleotide specific probes as described previously [23]. Evidence for *RET/PTC3* or *RET/PTC1* rearrangement in the cells from a given flask was scored as one *RET/PTC* event. The experiments were performed in triplicates.

### Statistical analysis

Statistical analysis was performed using the IBM SPSS Statistics version 25 software package. Data were analyzed using *T*-test for two groups comparison or One-way ANOVA followed by Tukey-Kramer posttest for multiple group comparison. All P values were 2-sided and considered significant if less than 0.05. In the experiments on generation of *RET/PTC*, due to data structure, the comparisons between experimental groups were based on the estimates of 95% confidence intervals (CI). The differences between means that did not have an overlap of 95% CI were considered significant.

## SUPPLEMENTARY MATERIALS FIGURES



## Abbreviations

DSB: DNA double strand break
NHEJ: non homologous end-joining
HDR: homology-directed repair
ALT: alternative lengthening of telomeres
NMI: nuclear myosin I
FISH: fluorescence in situ hybridization
4-OHT: 4-hydroxytamoxifen
