# Supplementary material for: Nuclear myosin/actin-motored contact between homologous chromosomes is initiated by ATM kinase and homology-directed repair proteins at double-strand DNA breaks to suppress chromosome rearrangements
Source: Oncotarget. 2018 Feb 7;9(17):13612–22. doi: 10.18632/oncotarget.24434 (PMC5862602; doi:10.18632/oncotarget.24434)
Supplement: Supplementary file 1 [file oncotarget-09-13612-s001.pdf]

Nuclear myosin/actin-motored contact between homologous chromosomes is initiated by ATM kinase and homology-directed repair proteins at double-strand DNA breaks to suppress chromosome rearrangements

SUPPLEMENTARY MATERIALS

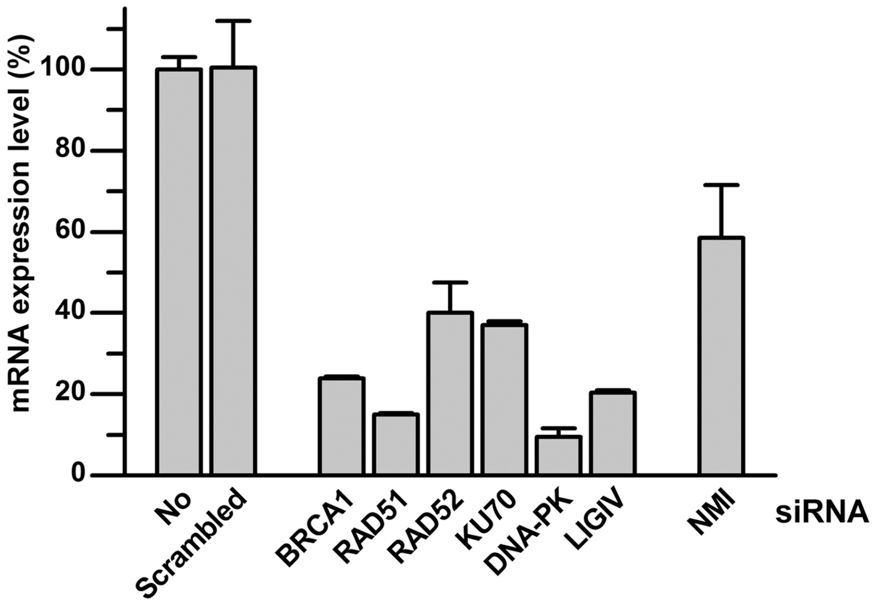

Supplementary Figure 1: Knockdown of HDR pathway (BRCA1, RAD51, RAD52), NHEJ pathway (KU70, DNA-PK, LIGIV) or nuclear myosin I (NMI) genes expression in TPC1/I-PpoI cells using siRNA. qRT-PCR was performed after treatment of TPC1/I-PpoI cells with specific siRNAs. Data are presented as mean ± SEM.

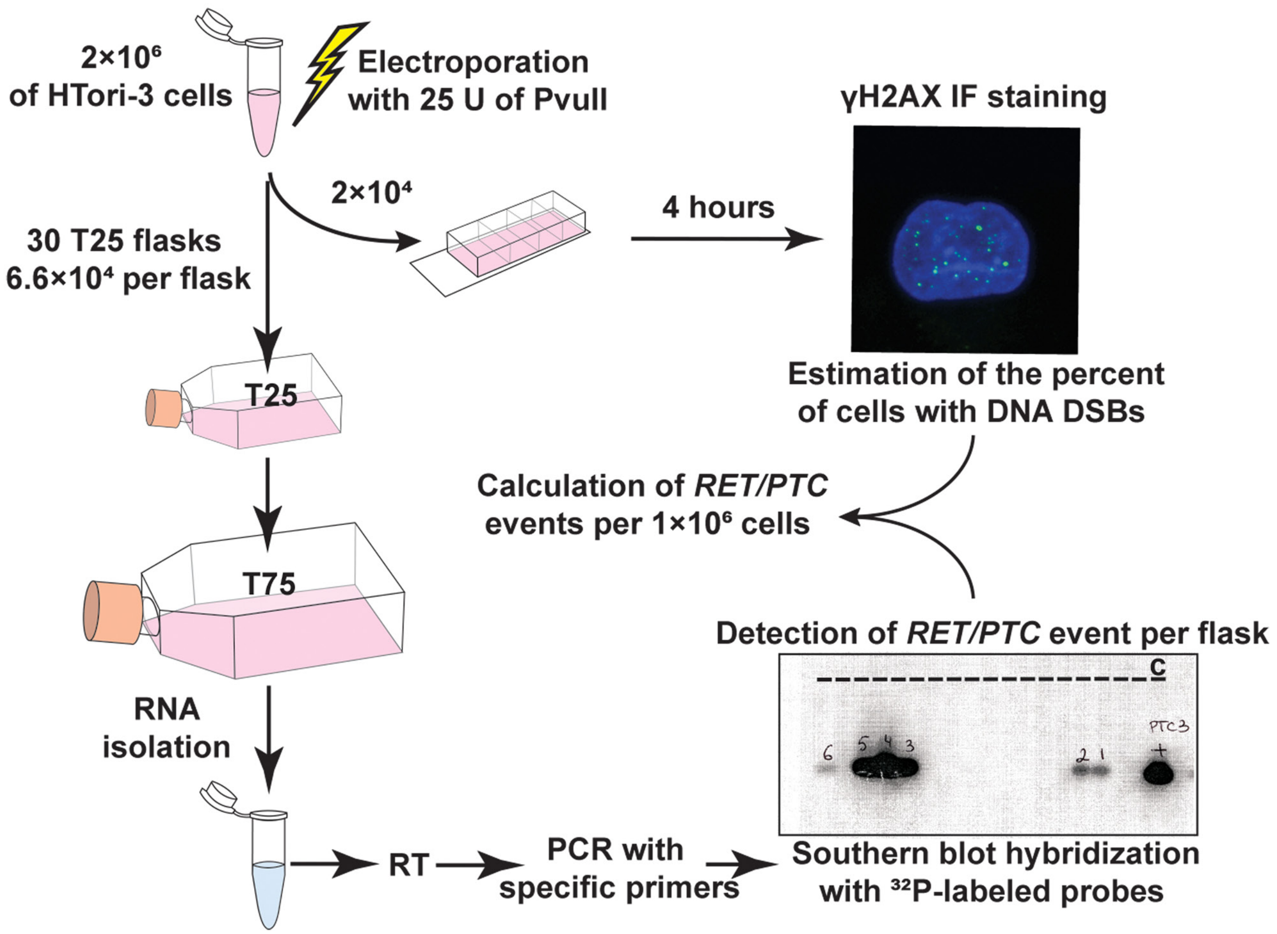

Supplementary Figure 2: Scheme of the experiment of *RET/PTC* rearrangements detection after induction of DNA double-strand breaks by PvuII.
